# Association between systemic immune-inflammation index and insulin resistance and mortality

**DOI:** 10.1038/s41598-024-51878-y

**Published:** 2024-01-23

**Authors:** Xiaoqi Deng, Dichuan Liu, Miao Li, Jie He, Yufan Fu

**Affiliations:** 1 Department of Cardiology, The Second Affiliated Hospital of Chongqing Medical University, Chongqing, 400010 China; 2 Nursing Department, Beijing Tiantan Hospital, Beijing, 100070 China

**Keywords:** Biomarkers, Diseases, Endocrinology, Risk factors

## Abstract

The role of inflammation in disease promotion is significant, yet the precise association between a newly identified inflammatory biomarker and insulin resistance (IR) and mortality remains uncertain. We aim to explore the potential correlation between systemic immune-inflammation index (SII) and these factors. We used data from 2011 to 2016 of National Health and Nutrition Examination Survey, and multivariate logistic regression and restricted cubic spline were employed. Subgroup and interaction analysis were conducted to recognize the consistency of the results. The association between SII and mortality was described by survival analysis. 6734 participants were enrolled, of whom 49.3% (3318) exhibited IR and 7.02% experienced mortality. Multivariate logistic regression revealed that individuals in the highest quartile (Q4) of SII had a significantly increased risk of IR compared to those in the lowest quartile (Q1). We then identified a linear association between SII and IR with an inflection point of 407, but may be influenced by gender. Similarly, compared to Q1, people whose SII at Q4 showed a higher all-cause and cardiovascular mortality. It showed a significant association between SII and both all-cause and cardiovascular mortality, but the results need to be interpreted with caution.

## Introduction

Insulin resistance (IR) is a multifaceted metabolic disorder characterized by intricate pathophysiological mechanisms, widely acknowledged as a prominent etiological factor in various diseases^1^. The development of IR is closely intertwined with internal environmental factors, including obesity^2^, substance metabolism^3^, and inflammation^4^, which collectively contribute to pathological alterations within the body. Among them, inflammation plays a crucial role in the pathophysiology of insulin resistance, obesity, and obesity-mediated insulin resistance^5,6^. However, obtaining traditional inflammation factor (such as TNF-a, interleukin-1) data is costly in clinical settings and poses challenges for scientific research and dissemination.

Systemic immune-inflammation index (SII), a new inflammatory biomarker based on platelet count × Neutrophil count/lymphocyte count calculation^7,8^, was proved to reflect the degree of systemic inflammation^9^. It reported that SII is independently associated with multisystem diseases, such as cardiovascular disease^10^, diabetic nephropathy^11^, rheumatic immune disease^12,13^, cancer^14–16^, osteoporosis^17^, metabolic diseases^18^. Different from traditional inflammatory factor, blood routine examination is convenient and inexpensive, and has higher practicability in disease research. Therefore, clarifying the relationship between systemic immune-inflammation index and insulin resistance could prove instrumental in advancing both scientific knowledge and clinical practice, leading to improved decision-making and reduced medical costs associated with treatment decisions. However, at present, there remains uncertainty surrounding this connection.

Therefore, we conducted a cohort study using the National Health and Nutrition Examination Survey (NHANES) database to investigate the impact of novel inflammatory markers on insulin resistance while exploring the effect of SII on mortality.

## Results

### Baseline characteristics of the participants

A total of 6734 participants were included in this study, of whom 49.2% were male, with an average age of 49.5 years. The prevalence of IR was 49.3% (3318), and the mean SII concentration was 496.9. The clinical characteristics of the participants were shown in Table 1, from which we can find that except for sex, smoking, renal failure, and stroke, the differences in other variables were statistically significant (*p* < 0.05). Participants with IR tended to be older, non-Hispanic white, alcohol drinkers, BMI ≥ 30, and abdominal obesity.

**Table 1:** Baseline characteristics of participants and distribution across IR.

	No-IR N = 3416	IR N = 3318	*P* value
Age (years)	47.86 ± 17.65	51.25 ± 16.97	< 0.001
HB (g/dL)	13.97 ± 1.51	14.14 ± 1.55	< 0.001
HBA1C (%)	5.48 ± 0.67	6.15 ± 1.44	< 0.001
FBG (mmol/L)	5.42 ± 0.90	6.76 ± 2.54	< 0.001
Sex (%)			0.178
Female	51.61	49.97	
Male	48.39	50.03	
Race (%)			< 0.001
Hispanic American	21.14	28.96	
Non-Hispanic White	41.80	36.65	
Non-Hispanic Black	19.50	21.01	
Non-Hispanic Asian	15.02	10.43	
Other/multi-racial	2.55	2.95	
Edu (%)			< 0.001
< 12th grade	20.73	25.17	
High school graduate	21.05	21.94	
Some college/AA degree	28.34	30.86	
College graduate or above	29.86	21.97	
PIR (%)			< 0.001
< 1.3	31.67	34.27	
1.3–4.9	49.15	51.21	
≥ 5	19.17	14.53	
Marriage^a^ (%)			< 0.001
Married	50.91	53.53	
Widowed	6.70	7.72	
Divorced/separated	13.09	14.38	
Never married	20.43	16.82	
Living with partner	8.84	7.54	
Drinking status (%)			< 0.001
No	25.73	30.38	
Yes	74.27	69.62	
Smoking status (%)			0.832
No	56.79	56.06	
Yes	43.09	43.82	
BMI^b^ (kg/m^2^) (%)			< 0.001
< 25	47.83	10.52	
25–29.9	34.78	29.60	
≥ 30	16.51	58.86	
Hypertension (%)			< 0.001
No	75.29	62.75	
Yes	22.10	34.54	
Diabetes mellitus (%)	0.00	0.00	< 0.001
No	89.08	64.17	
Yes	10.89	35.81	
Hypoglycemic drugs (%)			< 0.001
No	93.44	80.29	
Yes	6.56	19.71	
Hyperlipemia (%)			< 0.001
No	46.87	28.51	
Yes	53.13	71.46	
Liver disease (%)			< 0.001
No	96.75	94.06	
Yes	3.19	5.79	
Renal failure (%)			0.347
No	96.49	95.81	
Yes	3.43	4.07	
CHF (%)			< 0.001
No	97.31	95.36	
Yes	2.61	4.52	
CHD (%)			< 0.001
No	96.83	94.61	
Yes	2.93	4.94	
Stroke (%)	0.00	0.00	0.053
No	96.84	95.78	
Yes	3.07	4.16	
Cancer (%)			0.026
No	91.92	90.05	
Yes	8.05	9.89	
Abdominal obesity (AO, %)			< 0.001
No	63.88	22.97	
Yes	36.12	77.03	
SII (%)			< 0.001
Q1	27.17	22.39	
Q2	26.43	24.47	
Q3	24.50	25.26	
Q4	21.90	27.88	

*HB* hemoglobin, *HBA1C* glycosylated hemoglobin, *FBG* fasting glucose (mmol/L), *Edu* educational level, *PIR* ratio of family income to poverty level, *BMI* body mass index, *CHF* chronic heart failure, *CHD* coronary heart disease, *SII* systemic immunity-inflammation index.

^a^Marital status as of the time of inspection.

^b^BMI was calculated as weight (kg) divided by height squared (kg/m^2^).

### The relationship between SII and IR

Weighted multivariate logistic regression analysis was performed in Table 2, with SII dividing into quartiles (Q1–Q4). Compared to Q1, participants in Q4 were associated with an increased risk of IR, and the relationship was statistically significant in all 3 models. This association was significant in model 1 (OR 1.73; 95% CI 1.44–2.80), model 2 (OR 1.75; 95% CI 1.44–2.10), and model 3 (OR 1.42; 95% CI 1.14–1.77).

**Table 2:** Multiple logistic regression between SII and IR.

	Model 1		Model 2		Model 3	
	OR (95%CI)	*P*	OR (95%CI)	*P*	OR (95%CI)	*P*
SII						
Q1	Re	–	Re	–	Re	–
Q2	1.19 (0.99–1.43)	0.06	1.19 (0.99–1.43)	0.012	1.17 (0.94–1.45)	0.155
Q3	1.37 (1.13–1.64)	0.001	1.38 (1.15–1.67)	< 0.001	1.16 (0.93–1.45)	0.179
Q4	1.73 (1.44–2.80)	< 0.001	1.75 (1.44–2.10)	< 0.001	1.42 (1.14–1.77)	0.002
*P* for trend	< 0.001		< 0.001		0.018	

Model 1, no covariates were adjusted; Model 2 adjusted for age, sex and race. Model 3, age, sex, race, educational level, PIR, marital status, drinking status, smoking status, BMI, hypertension, diabetes mellitus, hypoglycemic agent, hyperlipemia, liver disease, renal failure, CHF, CHD, stroke, cancer, abdominal obesity, HB, HBA1C and FBG were adjusted.

**Figure 1:**
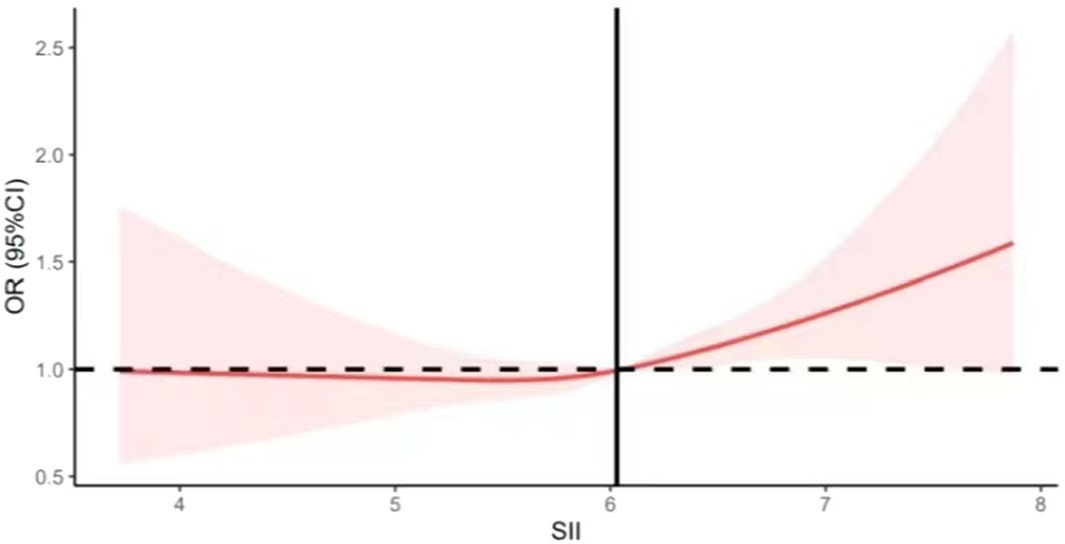
The relationship between SII and IR. After adjusting for multiple variables, we found a linear relationship between SII and IR with a threshold effect by using restricted cubic splines.

**Figure 2:**
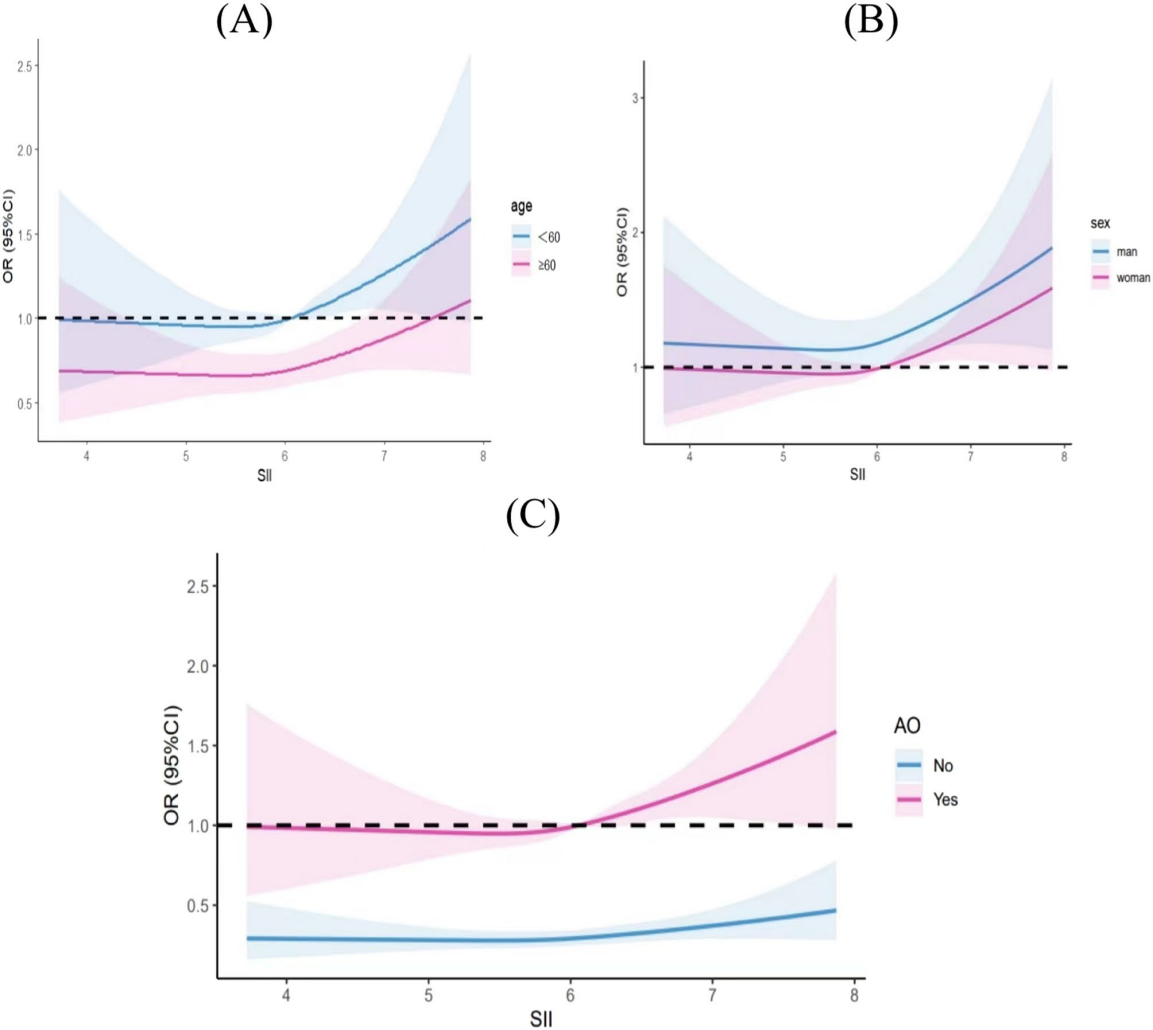
Results of restricted cubic spline according to sex, age, and abdominal obesity. (**A**)The smooth curve fitting result between systemic immune-inflammation index and insulin resistance in different genders. (**B**) The smooth curve fitting result between systemic immune-inflammation index and insulin resistance in different age. (**C**) The smooth curve fitting result between systemic immune-inflammation index and insulin resistance according to abdominal obesity.

After adjusting for multiple variables, we found a linear relationship between SII and IR by using restricted cubic splines (*p* = 0.37). As shown in Fig. 1, we can observe the threshold effect: that is, when LnSII was lower than 6.01 (SII = 407.5), the risk of IR is almost unchanged or even has a tendency to decrease; when LnSII exceeds 6.01, the risk of IR increases rapidly. Further stratification analyses (Fig. 2) showed that the intercept of the fitting curve was greater than 1 for man. Similar threshold effect was observed in age-stratified analysis. There was no relationship between SII and IR in non-abdominal obese subjects, while the inflection point of SII in abdominal obesity participants was 6.02 (SII = 411.6).

### The relationship between SII and mortality

**Figure 3:**
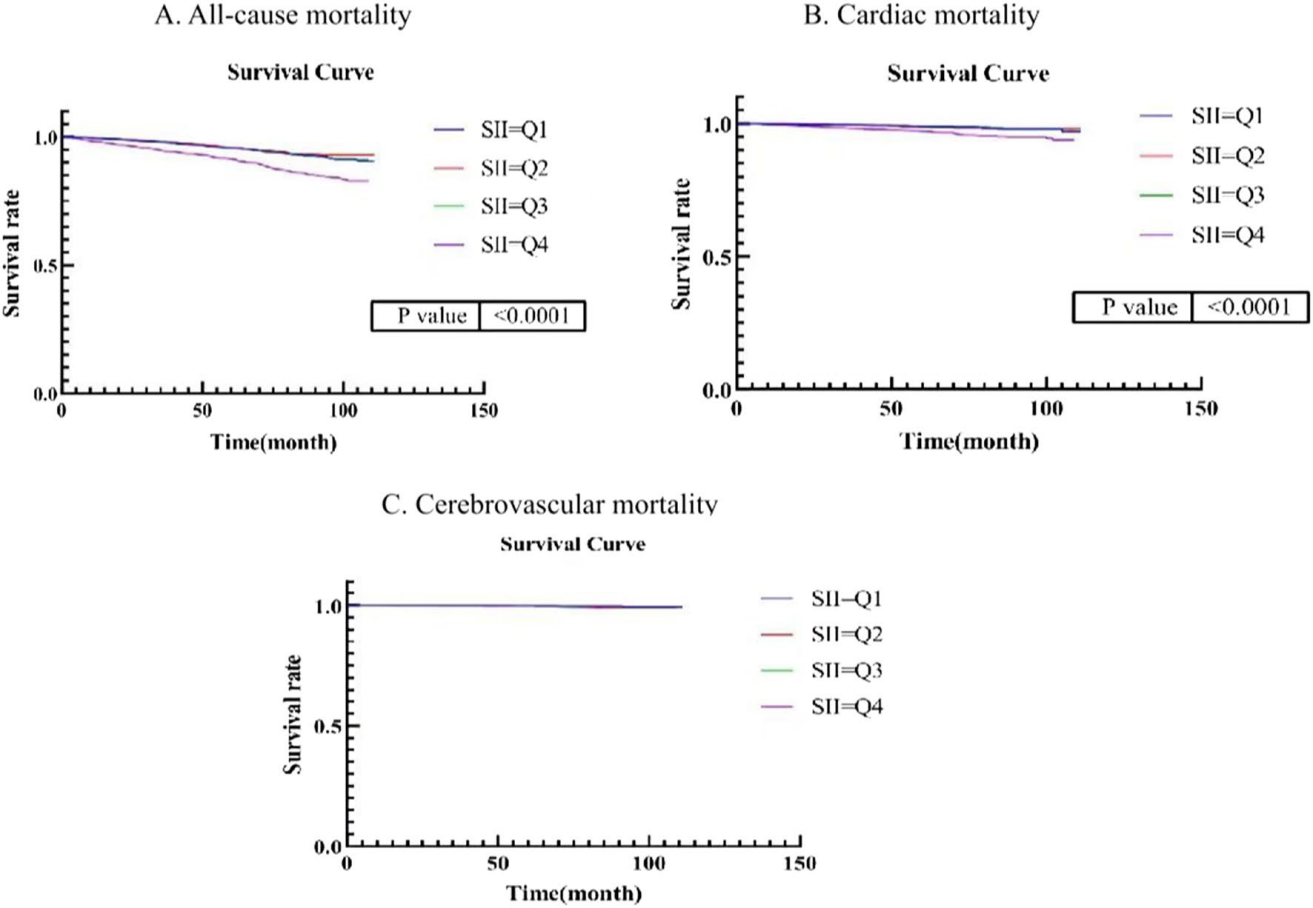
Kaplan–Meier survival curves for all-cause death, cardiovascular death, and cerebrovascular death. After grouping SII according to the quartile, COX regression analysis was performed to explore the relationship between SII level and the risk of death according to different causes of death.

The NHANES dataset encompassed the collection of death follow-up data spanning from 1999 to 2019. Within this study, the mean duration of follow-up was determined to be 70 months. Notably, the rates of all-cause mortality, cardiovascular mortality, and cerebrovascular mortality were observed to be 7.02%, 1.86%, and 0.34%, respectively. To accurately assess the impact of SII on mortality, survival analysis was conducted, wherein adjustments were made for intermediate factors. The selection of mediating variables was guided by a directed acyclic graph (DAG), which is visually presented in Supplementary Fig. 1. After excluding the potential mediating factors (insulin resistance, cancer, liver disease, and renal failure), the results of multivariate Cox regression models (as shown in Table 3) indicate a significant association between SII at the Q4 level and both all-cause mortality and cardiovascular mortality, when compared to the Q1 level. The hazard ratios for these associations were 1.33 (95% CI 1.03–1.72) and 1.79 (95% CI 1.07–3.00), respectively. However, no significant association was found between SII and cerebrovascular death (HR 1.54, 95% CI 0.43–5.61). However, due to the limited number of cerebrovascular death events (N = 23), the results need to be interpreted with caution. According to the findings depicted in Fig. 3, the Kaplan–Meier curves indicated a progressive increase in the all-cause mortality risk for individuals at the Q4 level as time progresses. Conversely, within the Q4 group, there was no apparent rise in the risk of cardiovascular death and brain death. To address model heterogeneity, we carried out a systematic examination of Model 1, 2, and 3 through progressive analyses, which demonstrated consistency across these iterations. Supplementary Table 1 presents these results, confirming the robustness of our survival estimates.

**Table 3:** Multiple Cox regression between SII and death.

	All-cause death		Cardiac death*		Cerebrovascular death^#^	
	HR (95%CI)	*P*	HR (95%CI)	*P*	HR (95%CI)	*P*
Q1	Re	–	Re	–	Re	–
Q2	0.63 (0.93–1.69)	0.634	0.95 (0.53–1.71)	0.859	1.79 (0.48–6.66)	0.384
Q3	0.87 (0.56–1.16)	0.338	0.80 (0.44–1.47)	0.479	1.99 (0.55–7.18)	0.291
Q4	1.33 (1.03–1.72)	0.029	1.79 (1.07–3.00)	0.027	1.54 (0.43–5.61)	0.509

*The diagnoses were based on ICD-10, which specifically included 100–109, 111, 113, 120–151.

^#^The diagnoses were based on ICD-10, which specifically included 160–169.

### Subgroup and interaction analysis

Subgroup analysis revealed that the association of SII and IR was not consistent in some groups. Overall, the relationship was statistically significant for participants who were female, younger than 60 years, non-Hispanic white, non-Hispanic black, and owned an abdominal obesity (*p* > 0.05). Furthermore, the interaction test showed that except for sex (*p* = 0.007), age, race, BMI, and abdominal obesity had no significant effect on the association (Table 4, interaction all *p* > 0.05).

**Table 4:**
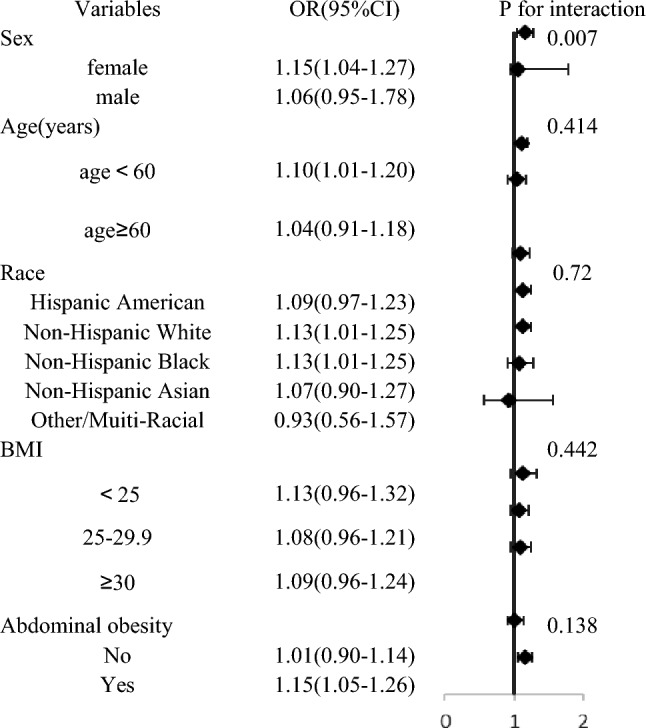
Subgroup and interaction analysis.

## Discussion

Our study harnesses the rich resources of the NHANES dataset to explore potential links between SII and clinical outcomes, specifically focusing on its relation to insulin resistance. Utilizing easily accessible and cost-effective blood tests, our findings may lead to informed decision-making, improving patient outcomes and reducing healthcare expenses associated with management strategies.

. In this cross-sectional study, we observed a positive linear association between higher SII and increased risk of insulin resistance. Notably, the risk of insulin resistance significantly escalated when SII exceeded 407.5. Further analysis using restricted cubic splines revealed that males and individuals younger than 60 years old were more susceptible to developing insulin resistance. Additionally, similar threshold effects were observed in individuals with abdominal obesity, while those without abdominal obesity exhibited lower susceptibility to insulin resistance, thus emphasizing the multifaceted benefits of weight management. Multiple Cox regression revealed a significant association between SII and both all-cause mortality and cardiovascular mortality. However, survival analysis demonstrated a lack of significant correlation between the SII and cardiovascular death, necessitating cautious interpretation of these results. Subgroup analysis indicated an interaction effect between sex and SII; however, further prospective studies are warranted for confirmation. Furthermore, a multivariate logistic regression excluding sex demonstrated that compared to the first quartile (Q1), individuals in the fourth quartile (Q4) had a higher odds ratio of 1.48 (95% CI 1.19–1.84) for developing insulin resistance—this finding was consistent with our previous result (OR 1.42; 95% CI 1.14–1.77).

To the best of our knowledge, previous studies have demonstrated a correlation between insulin resistance and inflammation. Initial investigations reported elevated levels of fibrinogen and other acute phase reactants in the bloodstream of individuals with insulin resistance^19,20^. It is noteworthy that several pro-inflammatory cytokines have been implicated in IR. For instance, Hotamisligil and Karasik were the first to identify TNF-α as an inducer of insulin resistance^21,22^. Animal experiment have shown that both classical IL-6 signal and IL-6 trans-signal can promote inflammation and insulin resistance^23^. An observational study revealed a positive association between high levels of IL-1 receptor antagonist and IR among African-Americans^24^. In target organs such as the liver and muscle tissue, macrophage polarization plays a pivotal role in the progression of IR^25^, thereby influencing both metabolism and inflammation through interactions with macrophages^26^. Notably, our stratified analysis on abdominal obesity indicated an intercept greater than 1 when SII was zero, suggesting that obesity may independently contribute to the risk of developing insulin resistance. Furthermore, previous research has consistently highlighted the significant role played by chronic inflammation in obesity-related insulin resistance^27–29^, which aligns with our findings. A study investigating racial differences suggested that adiposity mediates the relationship between inflammatory markers and IR specifically among African Americans^30^. Consistent with this notion, intensive lifestyle interventions aimed at weight management have been shown to reduce markers associated with inflammation and coagulation within diabetes prevention programs^31^, further emphasizing their importance for promoting overall health.

Extensive research spanning over 6 decades has been dedicated to investigating the mechanism underlying insulin resistance, yet the fundamental pathogenic signal remains elusive^32^. Inflammation-induced insulin resistance may be influenced significantly by Jun N-terminal kinase (JNK) and IκB kinase-β (IKKβ)/NF-κB. Classical receptor-mediated mechanisms involving proinflammatory cytokines such as TNF-α, IL-1, Toll, and AGE receptors activate JNK and IKKβ/NF-κB^4^. Cai^33^ reported that lipid accumulation leads to inflammation by activating NF-kB and inducing cytokine production, resulting in both local and systemic insulin resistance (IR). Additionally, the IKKβ/NF-κB axis has been identified as the target for salicylates and statins in the treatment of IR^34^. Extensive research has demonstrated a strong association between obesity-induced inflammation and IR^35,36^. The disorder of lipid metabolism results in the generation of inflammatory factors in various insulin target organs, which plays a crucial role in the development of IR^28^ and is considered a key characteristic of chronic inflammation associated with obesity^21,37^. Winer^38^ observed that diet-induced obesity led to a reduction in the expression of Ym1, arginase 1, and Il10 genes, causing a shift in the polarization state of macrophages from an anti-inflammatory M2 state to a proinflammatory M1 state in ATMs. Similarly, Patsouris found that the elimination of CD11c-positive cells resulted in a decrease in the expression of pro-inflammatory genes (such as macrophage and pro-inflammatory cytokine IL-6) and an increase in the expression of anti-inflammatory genes (such as anti-inflammatory cytokine IL-10) in ATMs, leading to the restoration of insulin sensitivity in obese insulin resistant mice^39^. Adiponectin, the most abundant protein secreted by adipose tissue, demonstrates significant anti-inflammatory effects through the activation of AMPK and PPAR-α signaling pathways via adiponectin receptor 1 (AdipoR1) and AdipoR2 respectively^40–42^. The downregulation of gene adiponectin occurs when there is a disruption in adipose tissue metabolism, resulting in reduced fatty acid oxidation and glucose uptake in muscle. Conversely, this disruption promotes gluconeogenesis in liver tissue, which is a secondary effect of IR. Amitan demonstrated that leptin, a substance secreted by adipocytes, can mitigate body weight and normalize blood glucose levels by binding to the leptin receptor (LepRb) and JAK2/STAT3^43^. Furthermore, certain antidiabetic agents possess anti-inflammatory properties that can enhance insulin resistance in individuals with diabetes. In a study involving older individuals, a physiological dose of metformin (100 M) was found to inhibit Th17 inflammation in CD4 T cells through an autophagy-dependent mechanism^44^. Furthermore, an animal experiment revealed that empagliflozin induces fat utilization and browning, reduces inflammation and insulin resistance in diet-induced obese mice by polarizing M2 macrophages^45^.

DAG was employed to mitigate the influence of mediating variables. However, the association between SII and cardiovascular death exhibited inconsistency when examined through proportional hazards regression model and survival analysis. This inconsistency may be attributed to the limitation of considering only a single variable in the survival analysis, neglecting potential confounding factors. Nevertheless, the Kaplan–Meier curve visualization demonstrated an upward trend in mortality risk for individuals in the Q4 level, warranting cautious interpretation of the findings. Although our results showed no statistically significant correlation between SII and cerebrovascular death, the limited sample size of cerebrovascular deaths in this study (n = 23) precludes a definitive conclusion regarding the association between SII and cerebrovascular mortality. To properly interpret these findings, future research with larger sample sizes is required. It is worth noting that our study fully accounted for the robust association between SII and all-cause mortality, aligning with the findings of a prior study. This study examined the link between SII and long-term mortality in patients with stroke-associated pneumonia, revealing a substantial association between SII and mortality^46^.

The subgroup analysis indicated that gender may have had an impact on the assessment of IR by the SII. This could be attributed to the potential influence of estrogen deficiency, as the average age of the participants in our study was 50 years. Estrogen has been found to inhibit β-cell apoptosis^47^, decrease pro-inflammatory signaling^48^, and enhance insulin function^49^. Studies have demonstrated that both animals and humans lacking endogenous estrogen production display insulin resistance, which can be mitigated through estrogen supplementation^50–52^. Furthermore, genetic studies have also confirmed that women are more prone to developing insulin resistance. The results of the animal experiment indicate that there is a higher expression of metabolic genes in male mice^53^. Additionally, the expression of genes involved in regulating protein synthesis in fuel metabolism, such as glucose and lipid oxidation, was found to be higher in males compared to females, suggesting that males may possess a greater capacity for substrate utilization^54^. Furthermore, the presence of polymorphisms in the scavenger receptor class B, member I (SCARB1), a gene associated with diabetes and regulated by estrogen, was found to be linked to insulin resistance, particularly in women^55^.

One notable advantage of this study is its extensive sample size and appropriate adjustment for covariates. Additionally, the inclusion of subgroup and sensitivity analyses enhances the reliability and comprehensiveness of the study findings. Nevertheless, it is important to acknowledge the limitations of our study. Firstly, the determination of the temporal order between SII and IR remains inconclusive. Secondly, the diagnostic process of the diseases relied on self-reported information provided by the respondents, thereby introducing the possibility of recall bias. Lastly, due to the inherent temporal aspect of disease recognition, potential influencing factors may not have been adequately accounted for.

## Materials and methods

### Study design

The NHANES is a national cross-sectional survey directed by the national center for health statistics of the Centers for Disease Control and Prevention to assess the health and nutritional status of adults and children in the United States. The survey examines a nationally representative sample of about 5000 people each year, and data are collected from a home interview and standardized physical mobile examination centers (MECs) released in 2-year cycles. The study was approved by the ethics review committee of the National Center for Health Statistics (NCHS) and obtained the written informed consent of each participant.

### Study population

**Figure 4:**
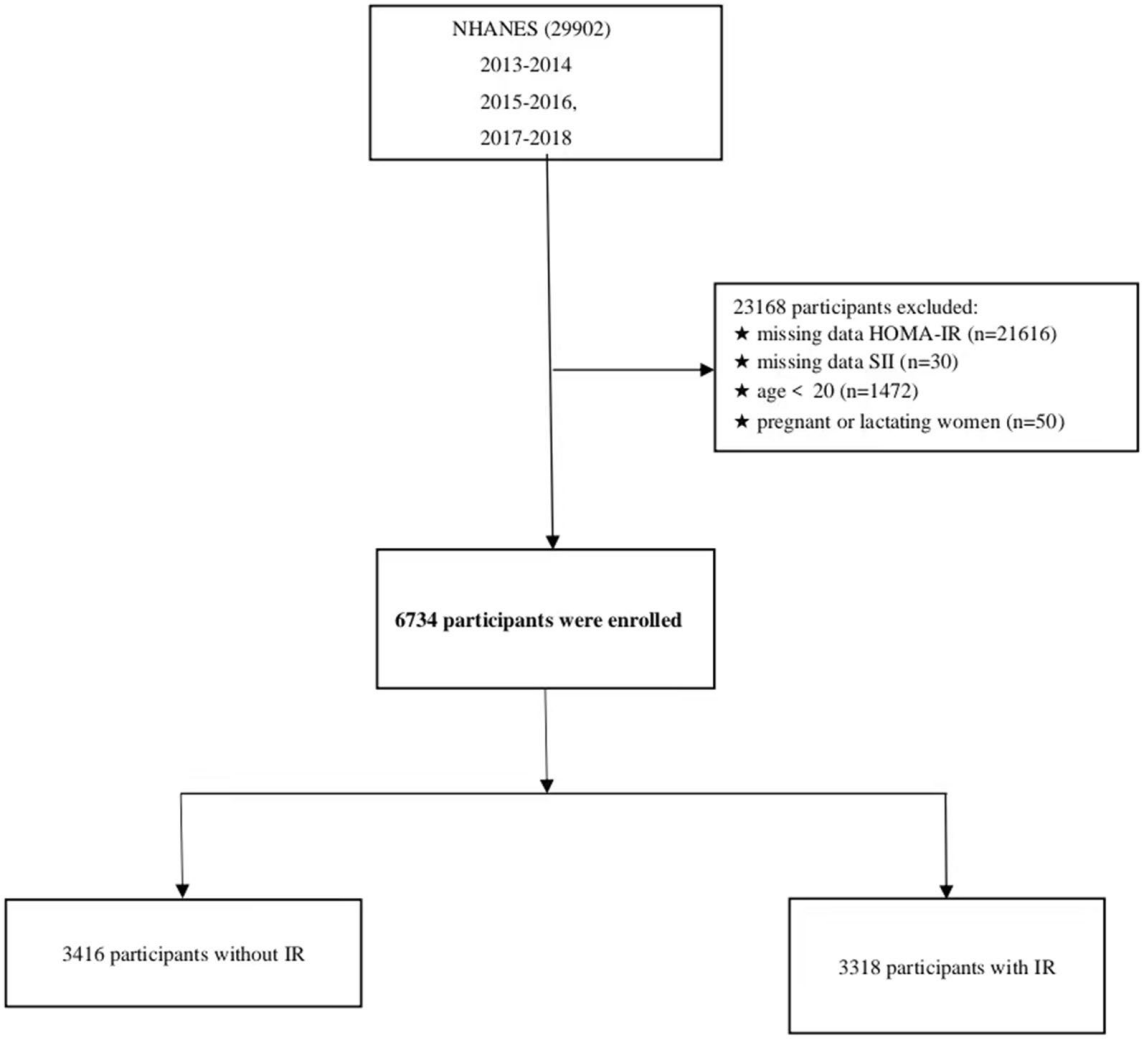
Flow diagram of the inclusion and exclusion criteria from 2013 to 2018 National Health and Nutrition Examination Survey (NHANES).

We used NHANES data from 2011 to 2016, which included 29,902 participants. Our study exclusions were as follows (Fig. 4): (1) individuals with missing fasting insulin or glucose measurements; (2) individuals with missing data of platelets, neutrophils, and lymphocyte; (3) adults aged less than 20 years old; (4) pregnant or lactating. A total of 6734 individuals were ultimately included in this study.

### Definition of systemic immune-inflammation

NHANES uses several methods to monitor the quality of the analyses performed in the MECs, and the results are measured in duplicate and averaged. The NHANES quality assurance and quality control (QA/QC) protocols meet the 1988 Clinical Laboratory Improvement Act mandates. The SII level was determined by multiplying the platelet count by the neutrophil count/lymphocyte count^56–58^. SII was ln-transformed when conducting regression analysis because of the right-skewed distribution of data.

### Definition of insulin resistance

Because of the limitations of the implementation condition of the euglycemic–hyperinsulinemic clamp method, previous studies used homeostatic model assessment (HOMA-IR) to assess IR, with the formula: HOMA-IR = fasting serum insulin (uIU/mL) × fasting plasma glucose (mmol/L)/22.5^59–61^. Evidence suggests that it is a strong correlation between IR estimation using HOMA-IR and the gold standard euglycemic-hyperinsulinemic clamp method^62^. IR was defined as a HOMA-IR ≥ 2.5, and non-insulin resistant as a HOMA-IR < 2.5^63,64^.

### Covariates

The covariates included in our study that may affect the RI include: age, sex, race (Hispanic American, Non-Hispanic White, Non-Hispanic Black, Non-Hispanic Asian and Other/Multi-Racial), educational level (below 12th grade, high school graduate, some college/AA degree or college graduate or above), marital status (married, widowed, divorced/separated, never married or living with partner), ratio of family income to poverty level (< 1.3, 1.3–4.9, ≥ 5), BMI (< 25.0 kg/m^2^, 25.0–29.9 kg/m^2^, ≥ 30 kg/m^2^), smoking status, alcohol consumption, diabetes mellitus, hypoglycemic agent consumption, hypertension, hyperlipemia, liver disease, renal failure, coronary heart disease (CHD), chronic heart failure (CHF), stroke, cancer, abdominal obesity (AO), hemoglobin (HB), glycosylated hemoglobin (HBA1c) and fasting blood-glucose (FBG). Trained staff used standardized techniques to measure waist circumference for all adults aged 20 years or older. Abdominal obesity was defined as a waist circumference more than 102 cm in men and 88 cm in women. Smoking status was obtained from the question “have you smoked at least 100 cigarettes in your entire life?” and individuals who answered “yes” would be enrolled in the study. Participants who drank more than 12 times in their lifetime were enrolled.

Hypertension was defined as the respondents' self-reported use of antihypertensive medications or an elevated mean of three blood pressure measurements: systolic blood pressure ≥ 140 mmHg and/or diastolic blood pressure ≥ 90 mmHg. Diabetes mellitus (DM) was defined as: (1) self-reported DM; (2) FBG ≥ 7.0 mmol/l, (3) 2-h postprandial blood glucose ≥ 11.1 mmol/L; (4) HBA1C ≥ 6.5 mmol/L; (5) taking hypoglycemic agents. Hyperlipemia was defined as total cholesterol ≥ 200 mg/dL, triglycerides ≥ 150 mg/dL, or low-density lipoprotein ≥ 130 mg/dL, and those who reported using cholesterol-lowering drugs were also classified as having hyperlipidemia. For the remaining comorbidities, participants who self-reported affirmations were considered to have the disease. For example, in the item “the doctor once told you that you had a stroke”, based on the responses, those who answered “1” were thought they had a stroke.

### Statistical analysis

Continuous variables were described as the mean ± SD and were compared by student’s t-test or Mann–Whitney U test, while categorical variables were described as percentages and were compared using chi-square test. Because the SII was unevenly distributed and clearly skewed to the right, we log-normalized the data for analysis. SII was divided into quartiles from lowest (Q1) to highest (Q4) to enhance the sensitivity. Multivariate logistic regression analysis was used to estimate the prevalence odds ratios (ORs) and 95% confidence intervals (CIs) between SII and IR. With model 1 including no covariates, model 2 was adjusted for age, sex and race and model 3 was further adjusted for educational level, PIR, marital status, drinking status, smoking status, BMI, hypertension, diabetes mellitus, hypoglycemic agent, hyperlipemia, liver disease, renal failure, CHF, CHD, stroke, cancer, abdominal obesity, HB, HBA1C and FBG. The restricted cubic splines were used to explore the non-linear relationships and inflection points. Multivariate tests were constructed by controlling for variables and fitting a smooth curve. The association between SII and mortality was analyzed by multiple Cox regression and survival analysis. Finally in sensitivity analyses, we performed subgroup and interaction analysis. Due to the complexity sampling survey of the NHANES database, we used appropriate weights for the analysis (WTMEC2YR/3). The statistical analyses were conducted using EmpowerStats (version 4.1), R studio (Version 4.3.1) and STATA (version 17.0). All reported probabilities (*P* values) were two-sided with *P* < 0.05 considered statistically significant.

### Ethics approval

The protocol for the research project has been approved by a suitably constituted Ethics Committee of the institution within which the work was undertaken and that it conforms to the provisions of the Declaration of Helsinki (as revised in Fortaleza, Brazil, October 2013).

### Approval of the research protocol

The study was conducted in accordance with the Declaration of Helsinki and was approved by the Institutional Review Board of the National Centre for Health Statistics (Protocol Number: #2011-17, #2018-01).

### Informed consent

All participants provided informed consent before enrollment.

### Supplementary Information

Supplementary Figure 1.

Supplementary Table 1.

## Data Availability

The datasets analysed during the current study are available in the [NHANES] repository, [https://www.cdc.gov/nchs/nhanes/index.htm].
