# Supplementary figures and images for: Association between systemic immune-inflammation index and insulin resistance and mortality
Source: Sci Rep. 2024 Jan 23;14:2013. doi: 10.1038/s41598-024-51878-y (PMC10806274; doi:10.1038/s41598-024-51878-y)

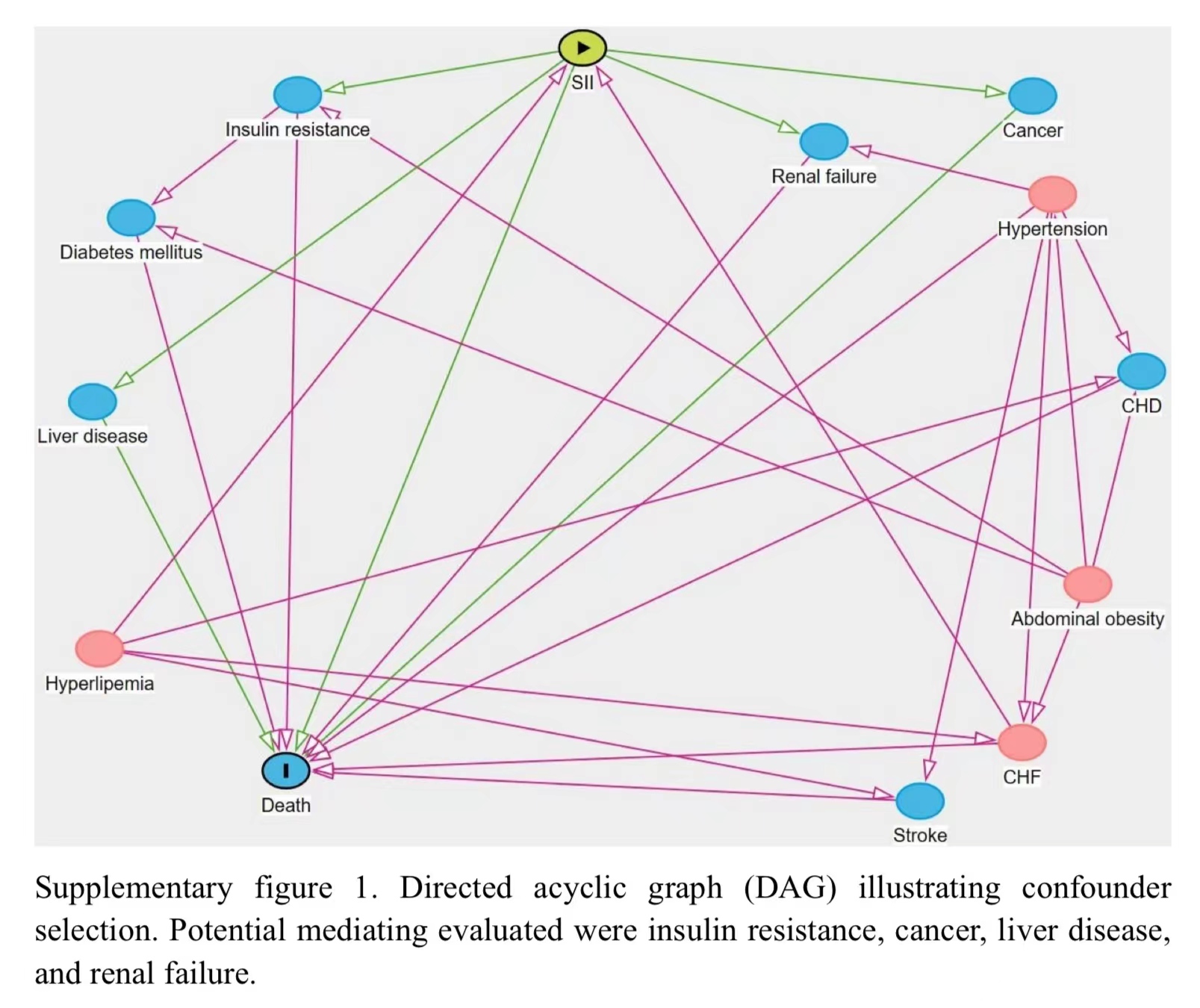

Supplement: Supplementary file 1 — Supplementary Figure 1. [file 41598_2024_51878_MOESM1_ESM.jpg]
